# The therapeutic efficacy of mesenchymal stromal cells on experimental colitis was improved by the IFN-γ and poly(I:C) priming through promoting the expression of indoleamine 2,3-dioxygenase

**DOI:** 10.1186/s13287-020-02087-7

**Published:** 2021-01-07

**Authors:** Ji-Young Lim, Byung-Su Kim, Da-Bin Ryu, Tae Woo Kim, Gyeongsin Park, Chang-Ki Min

**Affiliations:** 1 Department of Hematology, Seoul St. Mary’s Hospital, College of Medicine, The Catholic University of Korea, Seoul, Republic of Korea; 2 Department of Hematology, Eunpyeong St. Mary’s Hospital, College of Medicine, The Catholic University of Korea, 1021 Tongil Ro, Eunpyeong-gu, Seoul, 03312 Republic of Korea; 3 Department of Pathology, Seoul St. Mary’s Hospital, College of Medicine, The Catholic University of Korea, Seoul, Republic of Korea

**Keywords:** Mesenchymal stromal cells, Dextran sulfate sodium-induced colitis, Toll-like receptors, Indoleamine 2,3-dioxygen

## Abstract

**Background:**

Inflammatory bowel disease is a chronic and excessive inflammation of the colon and small intestine. We previously reported that priming of mesenchymal stromal cells (MSCs) with poly(I:C) induced them to express indoleamine 2,3-dioxygenase (IDO). We tried to find out whether the IFN-γ and poly(I:C)-primed MSCs have better therapeutic efficacy on the experimental colitis in the IDO1-dependent manner.

**Methods:**

To compare the therapeutic effects between the unstimulated MSCs and primed MSCs on murine colitis, mice (C57BL6) were administered with 2.5% dextran sodium sulfate (DSS) in drinking water for 5 days and injected with MSCs intraperitoneally on days 1 and 3 following DSS ingestion. The disease activity index score and body weight loss were assessed daily until day 9.

**Results:**

Mice receiving the IFN-γ and poly(I:C)-primed MSCs showed a reduced disease activity index and less weight loss. Colon tissue from the same mice presented attenuated pathological damage, increased Paneth cells, increased IDO1-expressing cells, and better proliferation of enterocytes. The primed MSC treatment upregulated the mRNA expression of intestinal stem cell markers (*Lgr5*, *Olfm4*, and *Bmi1*), enterocyte differentiation markers (*Muc2*, *Alpi*, *Chga*, and *occludin*), and regulatory T (Treg) cells (*Foxp3*). The same treatment decreased inflammatory cell infiltration to lymphoid organs and the level of pro-inflammatory cytokines (*IL-1β*, *TNF-α*, *IL-6*, and *MCP-1*) in colon tissue. Notably, in vivo pharmacologic inhibition of the IDO1 activity blocked the *Foxp3* upregulation in colon tissue and diminished the protective effects of the primed MSC.

**Conclusions:**

The priming of MSCs with the IFN-γ and poly(I:C) is a promising new strategy to improve the therapeutic efficacy of MSC and is worth further research.

## Background

Mesenchymal stromal cells (MSCs) have been tried to treat various inflammatory or auto-immune disorders since they are immune-modulatory, are not as immunogenic as other allogeneic cell types, and can contribute to tissue repair [1, 2]. They are easily isolated and expanded from bone marrow (BM) and even medical waste, such as adipose and umbilical tissues [3]. Their therapeutic efficacy on inflammatory bowel disease (IBD) was suggested by previous preclinical and clinical studies [4]. MSC injections improved the murine experimental colitis by downregulating Th1, Th17 responses [5, 6], upregulating Treg responses [7, 8], and inducing M2 macrophages [9]. MSCs secrete several soluble factors, including transforming growth factor-β (TGF-β), prostaglandin E2 (PGE2), nitric oxide (NO), and indoleamine 2,3-dioxygenase (IDO) to suppress activated T cells [10–12]. Secretion of these factors can be upregulated by pro-inflammatory cytokines, such as interferon (IFN)-γ, tumor necrosis factor (TNF)-α, and interleukin (IL)-1β [13]. Cell surface molecules, including programmed cell death-ligand 1 (PD-L1) and Fas ligand (FasL), are also induced by IFN-γ treatment and mediate the T cell suppression via cell-contact-dependent mechanisms [14, 15].

Recently, the allogeneic adipose-derived MSC product (Cx601) showed favorable outcomes in the phase III clinical trial and obtained the landmark approval in Europe for the treatment of complex perianal fistulas in Crohn’s disease [16]. Other studies also found the positive effects of locally injected MSCs, but they indicated that the efficacy decreased over time and that there was perhaps a need for repeated treatments [17]. In contrast to the success of local injection, systemic MSC therapy given intravenously for the luminal IBD has shown limited efficacy and inconsistent results [18]. More mechanistic studies in representative murine models of IBD are needed to bring the immune modulation by MSC to the clinic.

In our previous work, we primed murine BM-derived MSCs with various toll-like receptor (TLR) ligands and found that only the TLR3 ligand polyinosinic-polycytidylic acid [poly(I:C)] significantly increased the expression of IDO. Furthermore, the IFN-γ and poly(I:C)-treated MSCs could improve the pathologic scores of dextran sulfate sodium (DSS)-induced colitis more effectively than could unstimulated MSCs [19]. In the present study, we demonstrated that the IFN-γ and poly(I:C)-primed MSCs increased the Treg frequency, decreased inflammation, stimulated epithelial regeneration, and had better therapeutic efficacy on the murine IBD model than did unstimulated MSCs.

## Materials and methods

### Mice

Female C57BL/6 mice (9~10 weeks old) were purchased from Japan SLC, Inc. (Shizuoka, Japan). All animal experiments have been approved by the Institutional Animal Care and Use Committees of the Catholic University of Korea (Seoul, Republic of Korea).

### Priming of murine BM-derived MSCs

The BM-derived MSCs were isolated from C57BL/6 mice and expanded, as described in our previous work [19]. In brief, BM cells were flushed out from femurs and tibias, plated in 75 cm^2^ tissue culture flasks at a concentration of 1 × 10^6^ cells/mL in the complete culture medium, and incubated at 37 °C and 5% CO_2_. Non-adherent cells were removed after 3 days, and the remaining cells were passaged into a new flask when the cells reached 70~80% confluency. To do priming, we harvested cells at the 90% confluency and plated them in 12-well plates (5 × 10^4^ cells/well) in the complete culture medium supplemented with recombinant mouse IFN-γ (100 ng/mL, R&D Systems, Minneapolis, MN, USA). Poly(I:C) (TLR3 ligand, 10 μg/mL, Sigma-Aldrich, St. Louis, MO, USA) was added to the culture medium for stimulation. The primed MSCs were collected after 24 h and used for in vitro and in vivo experiments.

### DSS-induced murine colitis and MSC treatments

Experimental colitis was induced by administration of 2.5% DSS (molecular weight 36,000~50,000; MP Biomedicals, Santa Ana, CA, USA) in drinking water ad libitum for 5 days (day 0~5). Nine-week-old mice were randomly allocated into one of three groups: DSS only (DSS control), DSS with unstimulated MSC treatment (DSS + MSCs), and DSS with the treatment of MSCs stimulated with IFN-γ and poly(I:C) (DSS + primed MSCs). Unstimulated MSCs or the primed (stimulated) MSCs (3 × 10^6^ cells) were injected intraperitoneally (i.p.) on days 1 and 3. All mice were sacrificed on day 9 to harvest the spleen, mesenteric lymph node (mLN), and colon tissue. We daily assessed the severity of colitis until day 9 using body weight and the disease activity index (DAI), which is the summation of three parameters (1–4 score for each): body weight loss, stool consistency, and occult bleeding [20].

### L-1MT preparations

To prepare l-1-methyl tryptophan (L-1MT, Sigma-Aldrich, St. Louis, MO) for oral gavage, 1 g of L-1MT was added to a 15-mL conical tube with 7.8 mL Methocel/Tween [0.5% Tween 80/0.5% Methylcellulose (v/v in water; both from Sigma-Aldrich)]. The following day, the L-1MT concentration was adjusted to 85 mg/mL by adding an additional 4 mL Methocel/Tween and mixing again. For in vitro use, L-1MT was prepared as a 100-mmol/L stock in 0.1 N NaOH, adjusted to pH 7.4, and stored at − 20 °C protected from light.

### Hematoxylin-eosin (H&E) staining and immunohistochemistry (IHC)

We subjected formalin-fixed, paraffin-embedded tissue sections to H&E staining for microscopic examination. Slides were scored by a pathologist (blinded to experimental group). Pathologic severity of IBD was assessed by eight parameters: inflammatory infiltrate, goblet cell loss, crypt hyperplasia, crypt density, muscle thickness, submucosal infiltration, ulcerations, and crypt abscesses (0–3 score for each). A total histological severity score, ranging from 0 to 24, was obtained by summing the eight item scores [21].

Tissue sections (4 μm) were mounted on super frost glass sliders and deparaffinized in xylene and a graded series of ethanol, followed by antigen retrieval. Endogenous peroxidase activity was blocked with 3% hydrogen peroxide. Nonspecific binding sites were saturated by exposure to 10% normal goat serum diluted in phosphate-buffered saline (PBS) for 60 min. We incubated slides overnight at 4 °C with primary antibodies against mouse Ki-67 (1:100 dilution, Abcam, CB, UK), lysozyme (1:250 dilution, Abcam), and IDO1 (1: 400 dilution, Biolegend, San Diego, CA), then washed with PBS for 10 min. Biotinylated goat anti-rabbit IgG and rabbit anti-rat IgG (Vector Laboratories, Burlingame, CA) secondary antibodies were applied to tissue sections, and the slides were incubated at room temperature for 30 min. After the sections were washed and incubated for 30 min with peroxidase-conjugated streptavidin (Dako, Glostrup, Denmark) at room temperature, 3,3′-diaminobenzidine was added to visualize antigens. Sections were counterstained with Mayer’s hematoxylin, dehydrated, cleared, and mounted. We prepared negative control tissue samples in the same manner as described above, except that the primary antibody was omitted or replaced with an isotype control antibody (R&D Systems, Minneapolis, MN). IHC stains were evaluated for the presence of positively stained cells in 5 random fields under × 200 magnification on a light microscope (Leica DMI5000B, Germany). We counted the positively stained cells in each crypt.

### Quantitative RT-PCR

We isolated total RNA from colon homogenates with TRIzol® (Invitrogen, Carlsbad, CA, USA) according to the manufacturer’s instructions. One microgram of total RNA was reverse transcribed into cDNA. We did quantitative assessment of target mRNA levels by quantitative RT-PCR using a CFX96TM SYBR Green real-time PCR detection system (Bio-Rad, Hercules, CA, USA). The quantity of mRNA was calculated using the 2^–ΔΔCt^ method, and the level of β-actin was used to normalize total RNA quantities. The sequences of forward and reverse primers are shown in Table 1.

**Table 1:** Primers used for qPCR amplification

Gene	Forward sequence (5′-3′)	Reverse sequence (5′-3′)
Lgr5	ACCCGCCAGTCTCCTACATC	GCATCTAGGCGCAGGGATTG
OLFM4	TGGCCCTTGGAAGCTGTAGT	ACCTCCTTGGCCATAGCGAA
Bmi1	GCCACTACCATAATAGAATGTCT	TTGTGAACCTGGACATCACAAA
Axin2	GCAAACTTTCGCCAACCGTG	CTCTGGAGCTGTTTCTTACTGCCC
Muc2	GCTGACGAGTGGTTGGTGAATG	GATGAGGTGGCAGACAGGAGAC
Alpi	GGCTACACACTTAGGGGGACCTCCA	AGCTTCGGTGACATTGGGCCGGTT
Chga	AGGTGATGAAGTGCGTCCTG	GGTGTCGCAGGATAGAGAGGA
Occludin	GGACCCTGACCACTATGAAACAGACTAC	ATAGGTGGATATTCCCTGACCCAGTC
IL-1β	GCAACTGTTCCTGAACTCAAC	ATCTTTTGGGGTCCGTCAACT
TNF-α	GGAACACGTCGTGGGATAATG	GGCAGACTTTGGATGCTTCTT
IL-6	TCCATCCAGTTGCCTTCTTG	GGTCTGTTGGGAGTGGTATC
MCP-1	CTCACCTGCTGCTACTCATTC	GCTTGAGGTGGTTGTGGAAAA
Foxp3	ACAACCTGAGCCTGCACAAGTT	GCCCACCTTTTCTTGGTTTTG
IL-10	AGGGCCCTTTGCTATGGTGT	TGGCCACAGTTTTCAGGGAT
IDO1	ATTGGTGGAAATCGCAGCTTC	ACAAAGTCACGCATCCTCTTAAA
COX2	CCAGCACTTCACCCATCAGTT	ACCCAGGTCCTCGCTTATGA
PTEGS3	ATCACATGGGTGGTGATGAGGA	AGGCGATGACAACAGCCCTTAC
β-actin	AGCTGCGTTTTACACCCTTT	AAGCCATGCCAATGTTGTCT

*Lgr5* leucine-rich repeat-containing G protein-coupled receptor 5; *OLFM4* olfactomedin 4; *Bmi1* B cell-specific Moloney murine leukemia virus integration site 1; *Axin2* axis inhibition protein 2; *Muc2* mucin 2; *Alpi* alkaline phosphatase, intestinal; *Chga* chromogranin A; *IL-1β* interleukin 1 beta; *TNF-α* tumor necrosis factor alpha; *IL-6* interleukin 6; *MCP-1* monocyte chemoattractant protein 1; *Foxp3* forkhead box P3; *IL-10* interleukin 10; *IDO1* indoleamine 2,3-dioxygenase; *COX2* cyclooxygenase-2; *PTEGS3* prostaglandin E synthase 3

### Protein extractions and measurements of cytokines by ELISA

Colon samples were homogenized in 1 mL buffer solution (1× PBS, 1% NP40, 0.05% Na-deoxycholate, 0.1% SDS, and 1 tablet of Complete Protease Inhibitor Cocktail [Roche Diagnostics, Basel, Switzerland]), centrifuged at 3000 rpm for 20 min, after which supernatants were harvested. Total protein concentrations in supernatant were determined using the Bio-Rad Protein Assay (Bio-Rad, Hercules, CA). Concentrations of IL-1β, TNF-α, MCP-1, IL-6, and IL-10 were determined by ELISA using a commercially available kit (R&D systems, Minneapolis, MN, USA). Absorbance at 450 nm was measured using a microplate spectrophotometer, Benchmark Plus (Bio-Rad, Richmond, CA, USA).

### Flow cytometric analysis

Single-cell suspensions were stained in fluorescence-activated cell sorting (FACS) buffer at 4 °C for 30 min. We analyzed samples using an LSRII flow cytometer (BD Pharmingen, San Diego, CA). The following antibodies against mouse antigens were purchased from BD Pharmingen (San Diego, CA): BV605-conjugated anti-CD11b, FITC-conjugated anti-CD11c, BV450-conjugated anti-CD4, FITC-conjugated CD25, and PE-conjugated anti-Foxp3.

### Treg generation

We did the following experiments to analyze the effect of MSCs on T cell proliferation. We isolated untouched T cells from splenocytes using the Pan T Cell Isolation Kit (Miltenyi Biotec, Bergisch Gladbach, Germany). We co-cultured 2 × 10^5^ splenocytes with or without MSCs (primed or unstimulated) in the presence or absence of 2 μg/mL anti-CD3/CD28 (ebioscience, San Diego, CA, USA) plus 10 ng/mL recombinant murine TGF-β (R&D Systems) and 50 ng/mL recombinant murine IL-2 (R&D Systems) for 72 h. Then, T cells were harvested and surface stained for CD4, CD25, and Foxp3. A competitive IDO1 inhibitor, L-1MT (Sigma-Aldrich, St. Louis, MO, USA), was added to some wells.

### Statistical analysis

We performed statistical analyses using GraphPad Prism 7 software (GraphPad Software, Inc., La Jolla, CA, USA). All values are expressed as mean ± standard error of the mean. We did statistical comparisons between groups using the one-way ANOVA test with Bonferroni correction (post hoc). Differences were considered significant when the *p* value was less than 0.05.

## Results

### The IFN-γ and poly(I:C)-primed MSCs were more effective than unstimulated MSCs to ameliorate the DSS-induced colitis in mice

**Fig. 1:**
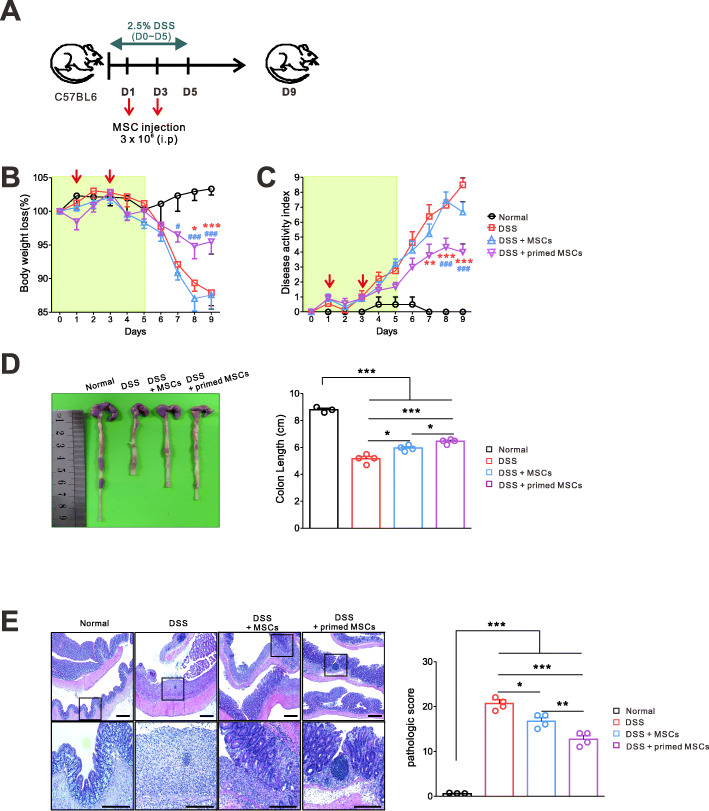
The IFN-γ and poly(I:C)-primed MSCs were more effective than were unstimulated MSCs in ameliorating the DSS-induced colitis in mice. **a** Experimental outline. 2.5% DSS was given to B6 mice via drinking water for 5 days to induce colitis. The primed or unstimulated MSCs were injected i.p. to mice on days 1 and 3. All mice were sacrificed on day 9 to harvest the spleen, mLN, and colon tissues. Results are combined from two independent experiments. **b** Daily body weight (normal, *n* = 4; other groups, *n* = 9). **c** Daily DAI score (DSS vs. DSS + primed MSC, *p < 0.05, **p < 0.01, ***p < 0.001; DSS + MSC vs. DSS + primed MSC, ^#^*p* < 0.05, ^##^*p* < 0.01, ^###^*p* < 0.001). **d** Colon lengths. **e** H&E-stained images of colon sections, representative data of two independent experiments, are shown (normal, *n* = 3; other groups, *n* = 4). The primed MSCs significantly reduced the pathologic scores (*p < 0.05, **p < 0.01, ***p < 0.001)

To identify the immunomodulatory effects of the IFN-γ and poly(I:C)-primed MSC, we used the murine DSS-induced colitis model (Fig. 1a). MSCs were primed with IFN-γ and poly(I:C) as described before [19] and injected i.p. to mice on days 1 and 3 (DSS + primed MSC group). Other mice were treated with the same volume of saline (DSS group) or unstimulated MSC (DSS + MSC group) on days 1 and 3. Compared to the DSS group and DSS + unstimulated MSC group, the DSS + primed MSC group had reduced DAI scores and less weight loss (Fig. 1b, c). Additionally, the primed MSC treatment shortened the colon length less, which is the anatomic marker of colonic inflammation (Fig. 1d). A pathologic examination confirmed the symptomatic improvement of the DSS-induced colitis, which was caused by the primed MSC. Figure 1e shows that the typical pathologic findings of the DSS-induced colitis (epithelial loss, crypt destruction, and inflammatory cell infiltration) were significantly ameliorated by the unstimulated MSC and further by the primed MSC. These results demonstrated the improved anti-inflammatory effects of the primed MSC compared to the unstimulated MSC.

### The IFN-γ and poly(I:C)-primed MSCs significantly stimulated the intestinal stem cell (ISC) proliferation, enterocyte differentiation, and epithelial regeneration

**Fig. 2:**
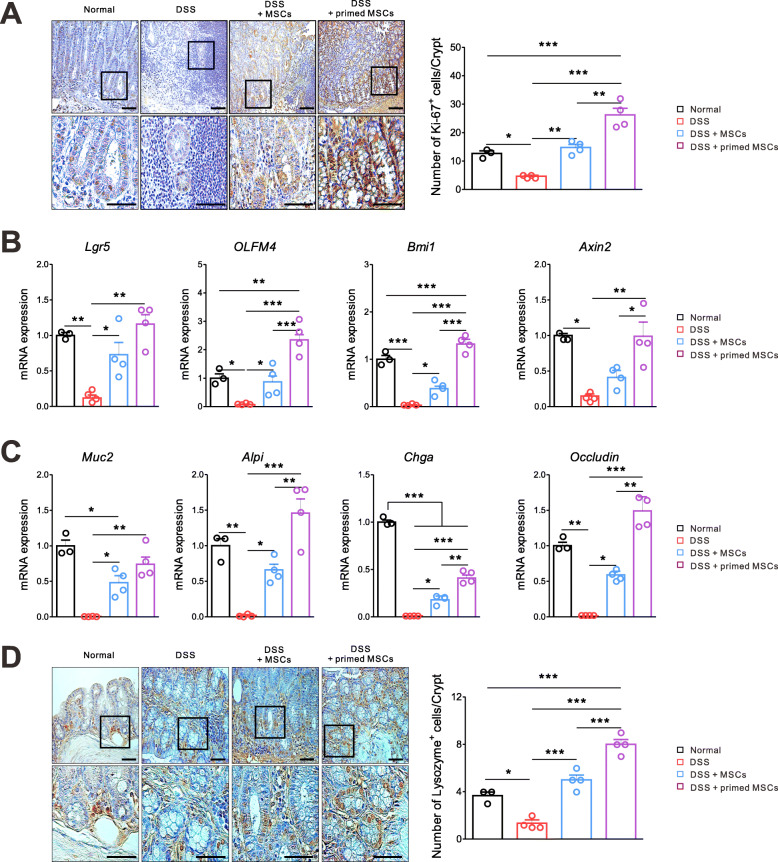
The IFN-γ and poly(I:C)-primed MSCs significantly stimulated the ISC proliferation, enterocyte differentiation, and epithelial regeneration. Mice with the DSS-induced colitis were sacrificed on day 9 to harvest colon tissues. Representative data of two independent experiments are shown (normal, *n* = 3; other groups, *n* = 4). **a** IHC images of colon sections for Ki-67 are presented. The Ki-67-positive cells in crypt are counted from the normal mice, the DSS control, the unstimulated MSC-treated, and the primed MSC-treated mice. **b** Results of quantitative PCR showed that mRNA levels of ISC markers (*Lgr5*, *Olfm4*, and *Bmi1*) and the Wnt/β-catenin pathway (*Axin2*) significantly increased in the primed MSC group. **c** The mRNA expressions of *Muc2* (goblet cell), *Alpi* (enterocyte), *Chga* (enteroendocrine cell), and Occludin (epithelial tight junction) significantly increased in the primed MSC group. **d** IHC images of colon sections for lysozyme are shown. The lysozyme-expressing Paneth cells in crypt are counted from the normal mice, the DSS control, the unstimulated MSC-treated, and the primed MSC-treated mice (*p < 0.05, **p < 0.01, ***p < 0.001)

We did the IHC staining of a cell proliferation marker, Ki-67, using colon tissues, which were harvested on day 9. The Ki-67 expression was prominently increased in the DSS + primed MSC group more than in other groups, suggesting the intestinal epithelial layer recovered significantly faster (Fig. 2a). Next, we investigated the ISC proliferation and Wnt/β-catenin signaling pathway, since they play a pivotal role in maintaining intestinal homeostasis [22]. Total RNA was isolated from colon tissues, and the mRNA of target genes was quantified by a real-time RT-PCR method. Markers of active ISC (*Lgr5* and *OLFM4*) and quiescent “4+” ISC (*Bmi1*) were significantly increased in the primed MSC group than in the DSS group and the unstimulated MSC group. The expression of *Axin2*, a target gene of the Wnt/β-catenin signaling pathway, was increased in the primed MSC group (Fig. 2b). The mRNA levels of *Muc2* (goblet cells), *Alpi* (enterocytes), *Chga* (enteroendocrine cells), and occludin (epithelial tight junction) were prominently upregulated in the primed MSC group more than in other groups (Fig. 2c). As compared with the normal control, the primed MSC group showed the significant upregulation of *Ki-67*, *OLFM4*, *Bmi1*, *ALPi*, and *Occludin*, but no difference in the expression of *Muc2* and the downregulation of *Chga*. This finding suggests that the primed MSC might be relatively better at restoring the intestinal epithelial barrier than inducing the differentiation of specific cell types. The IHC staining of lysozyme revealed that the primed MSC treatment significantly increased Paneth cells and preserved crypt structures better than did the unstimulated MSC (Fig. 2d). The number of Paneth cells was higher in the primed MSC group than the normal control. These results confirmed that the IFN-γ and poly(I:C) priming, i.e., TLR3 stimulation, increased the MSC’s efficacy in promoting intestinal epithelial regeneration and homeostasis than the unstimulated MSC.

### The IFN-γ and poly(I:C)-primed MSC decreased inflammatory cytokines in colon tissue and CD4^+^ T cells in lymphoid organs

**Fig. 3:**
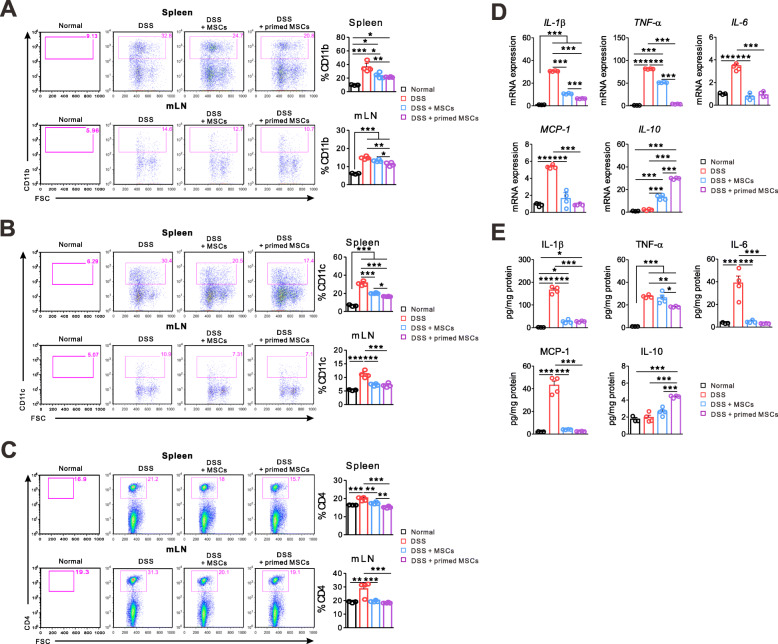
The IFN-γ and poly(I:C)-primed MSCs decreased inflammatory response in the spleen, mLN, and colon tissue. Mice with the DSS-induced colitis were sacrificed on day 9 to harvest the spleen, mLN, and colon tissues. Representative data of two independent experiments are presented (normal, *n* = 3; other groups, *n* = 4). **a**–**c** Recovered cells from the spleen and mLN were subjected to the flow cytometric analysis. The infiltration of monocytes (CD11^+^), dendritic cells (CD11c^+^), and CD4^+^ T cells significantly decreased in the primed MSC group compared to the DSS control. **d** Results of quantitative PCR from colon tissue demonstrated that mRNA expression of inflammatory cytokines (*IL-1β*, *TNF-α*, and *IL-6*) and a chemotactic factor (*MCP-1*) decreased significantly in the primed MSC group compared to the DSS control. In contrast, the expression of immune-modulating cytokine (*IL-10*) increased in the same group. **e** Protein is extracted from the colon tissue, and cytokine concentrations are measured by the ELISA assay (*p < 0.05, **p < 0.01, ***p < 0.001)

IBD is a chronic inflammatory state of the gastrointestinal tract, which involves effector T cells and inflammatory cytokines [1]. Therefore, we studied the anti-inflammatory and immunomodulatory effects of the primed MSC. Spleen, mLN, and colon tissues were harvested on day 9, as described in Fig. 1a. Like other findings, the primed MSC treatment significantly reduced the inflammatory cell infiltration into lymphoid organs compared to the DSS group. The numbers of monocytes (CD11b^+^), dendritic cells (CD11c^+^), and CD4^+^ T cells significantly decreased in the spleen and mLN from mice that had received the primed MSC treatment. The unstimulated MSC reduced the numbers of inflammatory cells in lymphoid tissues, except the monocytes in mLN, compared to the DSS group. The difference between the primed MSC and the unstimulated MSC was less prominent (Fig. 3a–c). Both types of MSC decreased the CD4^+^ T cells’ infiltration into the spleen and mLN down to the level of the normal mice, but the reduction of monocytes and dendritic cells by them did not reach the level of the normal mice, which implies that the MSC treatment might have limited efficacy on the myeloid cell infiltration into the lymphoid organs. Then, we performed the quantitative PCR assay to measure mRNA levels of cytokines in colon tissue. The primed MSC treatment significantly reduced the inflammatory cytokines (IL-1β, TNF-α, and IL-6) and increased the immune-modulating cytokine (IL-10) expression in the colon than did the DSS group. The unstimulated MSC treatment yielded intermediate results between the other two groups. The expression of IL-10 in the DSS group was as low as in the normal control, suggesting that immune-modulatory mechanism did not work for these mice. Notably, the primed MSC induced significantly higher expression of IL-10 and suppressed inflammatory cytokines more effectively than did the unstimulated MSC (Fig. 3d). The mRNA level of MCP-1, which recruits monocytes, dendritic cells, and T cells, was significantly lower in the colon tissue from mice that had received the primed or unstimulated MSC treatment. Still, the difference between these two groups was not significant (Fig. 3a–c). We also measured the cytokine concentrations in colon tissue by the ELISA assay and obtained the same results as the quantitative PCR (Fig. 3e). In summary, modulation of the cytokine profile in colon tissue was the most noticeable effect of the primed MSC. In contrast, their influence on myeloid cells in lymphoid tissue was significant but less evident.

### The IFN-γ and poly(I:C)-primed MSC expanded Treg cells in an IDO1-dependent manner

**Fig. 4:**
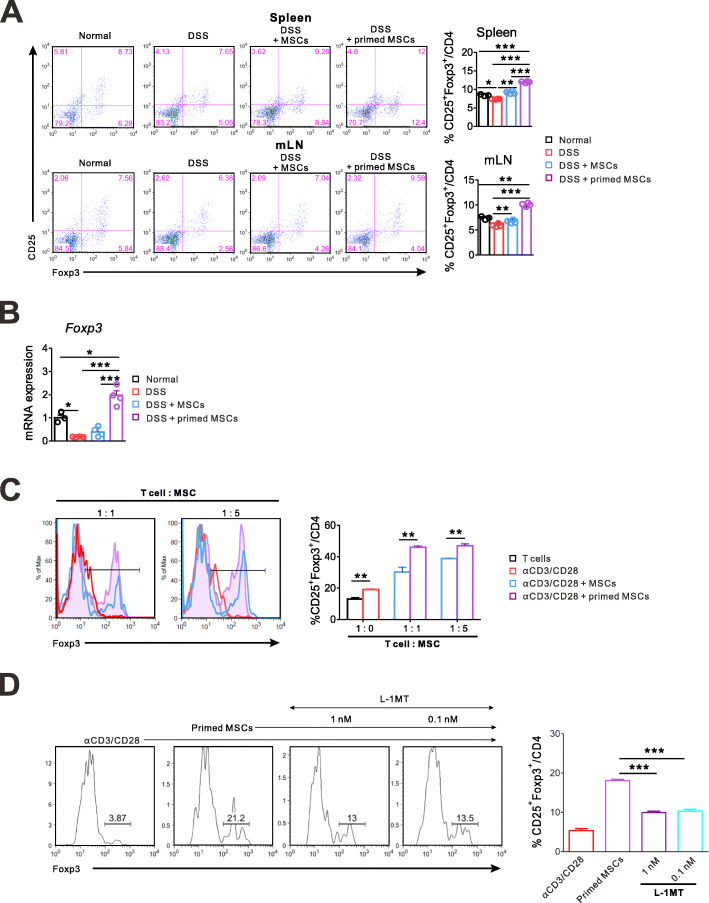
The IFN-γ and poly(I:C)-primed MSC expanded Treg cells in an IDO1-dependent manner. Mice with the DSS-induced colitis were sacrificed on day 9 to harvest the spleen, mLN, and colon tissue. Representative data of two independent experiments are presented (normal, *n* = 3; other groups, *n* = 4). **a** Treg (CD25^+^Foxp3^+^CD4^+^) proportions to CD4^+^ T cells in the spleen and mLN significantly increased in the primed MSC group than in the DSS control group. Difference of the Treg proportion between the primed MSC and the unstimulated MSC group was evident only in the spleen. **b** The colonic mRNA expression of Treg (*Foxp3*) markedly increased only in the primed MSC group. **c** T cells were negatively selected from splenocytes of B6 mice and co-cultured with the unstimulated or primed MSC in the presence of anti-CD3/CD28 antibodies. The primed MSCs increased the proportion of Treg (CD25^+^Foxp3^+^CD4^+^) to CD4^+^ T cells more effectively than the unstimulated MSC did. **d** A competitive IDO1 inhibitor, L-1MT, reversed the Treg expansion which was induced by the primed MSC (*p < 0.05, **p < 0.01, ***p < 0.001)

The Foxp3^+^ Treg cells were functionally defective, or their frequency was significantly lower in active IBD patients [23]. Interestingly, the anti-TNFα therapy, such as infliximab, significantly increased the frequency of functional Foxp3^+^ Treg cells in patients with active IBD [24]. In this study, we investigated the change of Foxp3^+^ Treg cell frequencies in lymphoid organs and colon tissue after treatments of the primed or unstimulated MSCs for DSS-induced colitis. The proportion of Treg (CD25^+^Foxp3^+^CD4^+^) to CD4^+^ T cells in the spleen was significantly higher in the primed MSC group than in the unstimulated MSC group and the DSS control group. Difference of Treg proportion between the primed and unstimulated MSC treatments was not prominent in mLN (Fig. 4a). The mRNA of *Foxp3* in colon tissue increased considerably more in the primed MSC group than in the other groups. The unstimulated MSC could not increase the *Foxp3* expression in colon tissue compared to the DSS control group, although they showed some anti-inflammatory effects on the IBD (Fig. 4b). In contrast, the primed MSC treatment significantly increased the Treg proportion in all three tissues than did the normal mice and the DSS control. We suppose that the ability of MSCs to induce the Treg cell expansion is the most important improvement induced by the IFN-γ and poly(I:C) priming. To further confirm the capacity of the primed MSCs for expanding Treg, we did in vitro experiments. In brief, T cells were isolated from splenocytes of B6 mice and co-cultured with the primed or unstimulated MSC. The primed MSC caused a significantly higher increase of Treg (CD25^+^Foxp3^+^) proportion to CD4^+^ T cells than did the unstimulated MSCs (Fig. 4c). Since we demonstrated that the IFN-γ and poly(I:C) priming of MSC induced the considerable IDO1 upregulation [19], we tried to find out whether the primed MSC promoted Treg cells in an IDO1-dependent manner. Figure 4d shows that a competitive IDO1 inhibitor, L-1MT, diminished the increment of Treg (CD25^+^Foxp3^+^) proportion induced by the primed MSCs, confirming our hypothesis.

### The IFN-γ and poly(I:C)-primed MSCs increased the expression of colonic IDO1 and COX2

**Fig. 5:**
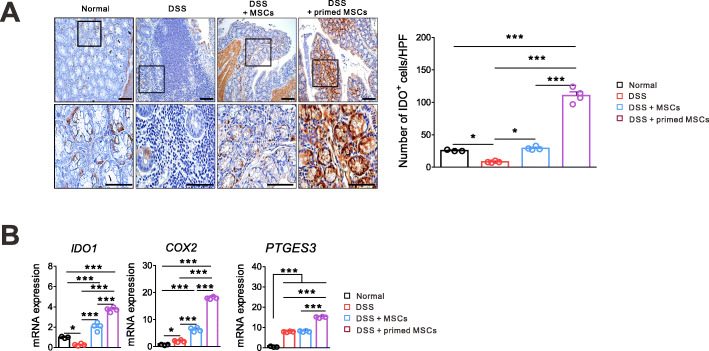
The IFN-γ and poly(I:C)-primed MSCs increased the expression of colonic IDO1 and COX2. Mice with the DSS-induced colitis were sacrificed on day 9 to harvest colon tissues. Representative data of two independent experiments are presented (normal, *n* = 3; other groups, *n* = 4). **a** IHC images of colon sections for IDO1 are shown. Quantification of IDO1-positive cells in the normal mice, the DSS control, the unstimulated MSC-treated, and the primed MSC-treated mice. **b** The colonic mRNA expression of *IDO1*, *COX2*, and *PTGES3* increased more in the primed MSC group than in other groups (*p < 0.05, **p < 0.01, ***p < 0.001)

We explored how the IDO1 and PGE2 pathways were affected by the MSC treatment since they promote intestinal homeostasis by limiting inflammatory responses and protecting the epithelium [25]. We did the IHC staining of IDO1 using colon tissues, which were harvested on day 9, as described in Fig. 1a. The primed MSC treatment induced a significant increment of IDO1-expressing cells in intestinal crypts than did the unstimulated MSCs, suggesting the role of IDO1 in maintaining the intestinal epithelial layer (Fig. 5a). The quantitative PCR confirmed the same finding for the IDO1 mRNA expression in colon tissue (Fig. 5b). We found that the primed MSCs induced a significantly higher expression of cyclooxygenase 2 (COX2) and prostaglandin E synthase 3 (PTGES3) genes in colon tissue than did the unstimulated MSC (Fig. 5b).

### Pharmacologic IDO1 inhibition decreased the therapeutic efficacy of the IFN-γ and poly(I:C)-primed MSCs on the DSS-induced colitis

**Fig. 6:**
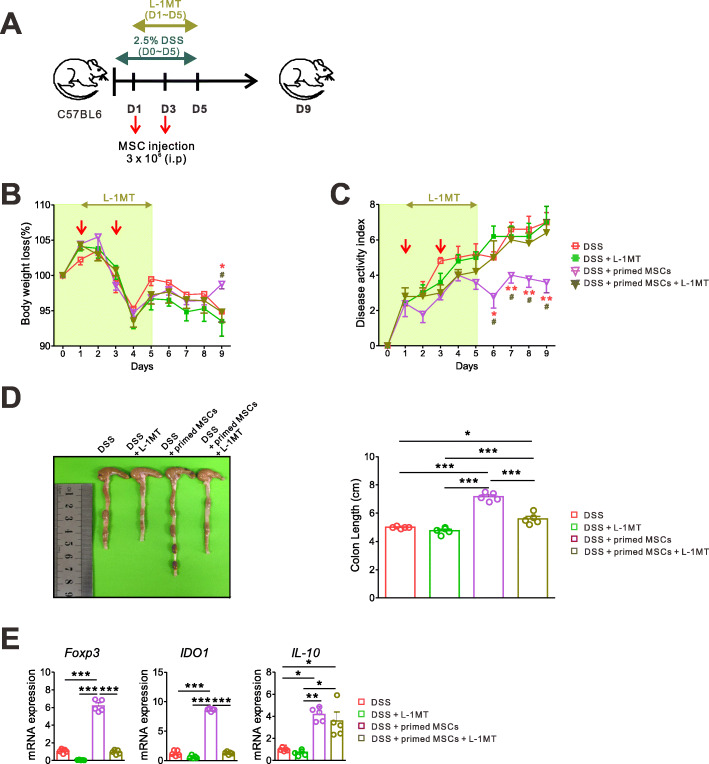
Pharmacologic IDO1 inhibition decreased the immune-modulatory effects of the IFN-γ and poly(I:C)-primed MSC. **a** Experimental colitis was induced, and the unstimulated or primed MSCs were administered as described in Fig. 1. The competitive IDO inhibitor, L-1MT (200 mg/kg), was administered by oral gavage from day 1 to day 5. All mice were followed up daily and sacrificed on day 9 to harvest colon tissues. Representative data of two independent experiments are presented (*n* = 5 for each group). **b** Daily body weight. **c** Daily DAI score (DSS vs. DSS + primed MSC, *p < 0.05, **p < 0.01, ***p < 0.001; DSS + primed MSC + L-1MT vs. DSS + primed MSC, ^#^*p* < 0.05, ^##^*p* < 0.01, ^###^*p* < 0.001). **d** Colon lengths were measured on day 9. **e** Results of quantitative PCR from colon tissue demonstrated that the oral L-1MT administration reversed the expression of *Foxp3* and *IDO1*, which was increased by the primed MSC treatment. Representative data of two independent experiments are presented (*n* = 5 for each group) (*p < 0.05, **p < 0.01, ***p < 0.001)

Next, we tried to find out whether IDO1 is essential for the protective effects of the primed MSCs. We induced experimental colitis and injected MSCs, as described in Fig. 1a. In addition, a competitive IDO inhibitor, L-1MT (200 mg/kg), was administered by oral gavage from day 1 to day 5 (Fig. 6a). We showed that the pharmacologic IDO1 inhibition abolished the beneficial effects of the primed MSC treatment, such as less weight loss, reduced DAI scores, and preserved colon length (Fig. 6b–d). Importantly, the mRNA expressions of *Foxp3* and *IDO1*, which were increased by the primed MSC treatment, were downregulated by the IDO1 inhibition. In contrast, the expression of *IL-10* was not affected by the IDO1 inhibition, implying that IL-10 was possibly secreted from monocytes rather than from Treg cells in the colon tissue of the experimental IBD, or that other mediators besides IDO1 might be involved in the IL-10 production (Fig. 6e). These results altogether suggest that IDO1 is an important mediator of the primed MSCs in promoting intestinal epithelial recovery and suppressing inflammatory responses.

## Discussion

Several clinical trials using MSCs have reported that they have been safe and effective treatments for IBD until now [16–18]. However, each trial used different procedures for tissue harvest, MSC isolation, and cell culture. The routes and timing of MSC administration were also diverse. This aspect made the obtained results frequently inconsistent and unimpressive [26]. The cell priming (also referred to as pre-conditioning) is one of the most studied approaches to overcome this problem and has been known to improve the immune-modulatory attributes of MSCs. Previous studies demonstrated that MSC priming with pro-inflammatory cytokines, such as IFN-γ and TNF-α, increased the secretion of anti-inflammatory cytokines (IDO1, PGE2, TGF-β, and hepatocyte growth factor), the expression of chemokine ligands (CXCL9, CXCL10, and CXCL11), adhesion proteins (VCAM-1 and ICAM-1), and immune checkpoint molecules, such as PD-L1 [27, 28]. In experimental colitis, human IFN-γ-primed MSCs showed higher migration rates to inflammatory sites and a significant reduction of mucosal damage and inflammatory responses than did non-primed MSC [29]. There is still some room for improvement in the IFN-γ priming of MSCs, because its effects can be inconsistent [30] and transient [31]. A few studies have been published regarding the priming of MSCs with TLR ligands, since TLRs are involved with the immune-modulating functions of MSCs [32]. Waterman and colleagues reported that TLR4-primed MSCs mostly elaborated pro-inflammatory cytokines, whereas TLR3-primed MSCs expressed mostly immunosuppressive mediators [33]. We previously showed that the priming of MSCs with a TLR3 ligand, poly(I:C), in the presence of IFN-γ increased IDO1 production and increased the immunomodulatory effects of MSCs. Other TLR ligands, Pam3CSK4 (TLR1/2), peptidoglycan (TLR2), LPS (TLR4), flagellin (TLR5), FSL-1 (TLR2/6), R848 (TLR7/8), and CpG (TLR9) did not increase IDO1 expression compared to IFN-γ alone [19]. Therefore, we hypothesized that the IFN-γ and poly(I:C) priming of MSCs could increase the therapeutic efficacy in an IDO1-dependent manner.

The upregulation of IDO1 is observed not only in the experimental murine colitis but also from the clinical samples of human IBD. CD103^+^ gut dendritic cells can express IDO1 and support Treg conversion while suppressing Th1/Th17 differentiation to limit gut inflammation [34]. In the homeostatic state, gut expression of IDO1 is low and limited to the cells of the lamina propria. However, the inflammatory cytokines, including IFN-γ, TNF-α, and IL-1β, stimulate the IDO1 expression in epithelial cells, which become a significant source of IDO1 activity in IBD. The IDO1 expression is more apparent in epithelial cells near the sites of ulceration [35]. In the experimental colitis model, the administration of the TLR-9 agonist improved clinical and histological parameters via the induction of IDO1, and the inhibition of IDO1 activity abrogated the protective effects [36]. Gurtner et al. also demonstrated that the administration of IDO1 inhibitor 1-DL-MT worsened the IBD activity in mice, suggesting that IDO1 downregulated Th1 responses within the intestinal tract [37]. The protective effects of IDO1 were confirmed in a mouse model of graft versus host disease. Jasperson et al. showed that IDO1^−/−^ mice exhibited greater colitis severity, T cell infiltration, and mortality [38]. They also demonstrated that induction of IDO1 primarily in professional APCs by a TLR-7/TLR-8 agonist reduced colon injury and decreased lethality [39]. IDO1 secreted from MSCs also polarized the differentiation of monocytes into IL-10-producing CD206^+^ M2 macrophages, which in turn promote T cell suppression [40]. In addition, IDO1 blocked the intestinal bacterial growth and mediated epithelial barrier protection induced by IL-27 [41]. It is worthy of note that IDO1 expression supported epithelial proliferation independently of the effects on adaptive immunity through the activation of the Wnt/β-catenin signaling pathway in the colitis-associated cancer model (azoxymethane/DSS) [42]. Taken together, IDO1 expression by APCs may be critical to suppressing inflammatory T cell responses, whereas epithelial IDO1 activity functions to limit microbial invasion and promote epithelial repair. Further studies are required to find out which one predominantly contributes to ameliorating IBD.

## Conclusion

In this study, we demonstrated that the IFN-γ and poly(I:C) priming had increased the therapeutic efficacy of MSCs on DSS-induced colitis. The primed MSCs alleviated the DSS-induced pathologic changes in the colon, decreased inflammatory cytokines, and stimulated the proliferation of ISC more effectively than the unstimulated MSCs did. Additionally, the primed MSCs stimulated the differentiation of intestinal epithelial cells and the restoration of the mucosal barrier to a greater extent. Last but not least, these improvements were mediated through the IDO1 and increased Treg proportion in the inflamed site. These results suggest that the priming of MSCs through TLR3 stimulation and IFN-γ is a promising new strategy to increase the therapeutic efficacy of MSC on IBD and might be able to solve the problems that the current MSC therapies are facing. IDO1 is a critical mediator of the IFN-γ and poly(I:C)-primed MSC in suppressing IBD, and further studies are required to unfold its functions in gut epithelium and the surrounding microenvironment.

## Data Availability

The data that support the findings of this study are available from the corresponding author upon reasonable request.

## Abbreviations

BM: Bone marrow
COX2: Cyclooxygenase 2
DAI: Disease activity index
DSS: Dextran sulfate sodium
FACS: Fluorescence-activated cell sorting
H&E: Hematoxylin-eosin
IBD: Inflammatory bowel disease
IDO: Indoleamine 2,3-dioxygenase
IFN: Interferon
IHC: Immunohistochemistry
IL: Interleukin
i.p.: Intraperitoneally
L-1MT: l-1-Methyl tryptophan
mLN: Mesenteric lymph node
MSC: Mesenchymal stromal cell
NO: Nitric oxide
PD-L1: Programmed cell death ligand 1
PGE2: Prostaglandin E2
PTGES3: Prostaglandin E synthase 3
TGF: Transforming growth factor
TLR: Toll-like receptor
TNF: Tumor necrosis factor
